# Essential Roles of the Tap42-Regulated Protein Phosphatase 2A (PP2A) Family in Wing Imaginal Disc Development of *Drosophila melanogaster*

**DOI:** 10.1371/journal.pone.0038569

**Published:** 2012-06-06

**Authors:** Ning Wang, Hung-Tat Leung, Matthew D. Mazalouskas, Guy R. Watkins, Rey J. Gomez, Brian E. Wadzinski

**Affiliations:** 1 Department of Pharmacology, Vanderbilt University Medical Center, Nashville, Tennessee, United States of America; 2 Department of Biological Sciences, Grambling State University, Grambling, Louisiana, United States of America; University of Iowa, United States of America

## Abstract

Protein ser/thr phosphatase 2A family members (PP2A, PP4, and PP6) are implicated in the control of numerous biological processes, but our understanding of the *in vivo* function and regulation of these enzymes is limited. In this study, we investigated the role of Tap42, a common regulatory subunit for all three PP2A family members, in the development of *Drosophila* m*elanogaster* wing imaginal discs. RNAi-mediated silencing of *Tap42* using the binary *Gal4/UAS* system and two disc drivers, *pnr-* and *ap-Gal4*, not only decreased survival rates but also hampered the development of wing discs, resulting in a remarkable thorax cleft and defective wings in adults. Silencing of *Tap42* also altered multiple signaling pathways (HH, JNK and DPP) and triggered apoptosis in wing imaginal discs. The *Tap42^RNAi^*-induced defects were the direct result of loss of regulation of *Drosophila* PP2A family members (MTS, PP4, and PPV), as enforced expression of wild type Tap42, but not a phosphatase binding defective Tap42 mutant, rescued fly survivorship and defects. The experimental platform described herein identifies crucial roles for Tap42•phosphatase complexes in governing imaginal disc and fly development.

## Introduction

PP2A, together with PP4 and PP6, constitute the PP2A family of phospho-ser/thr phosphatases, which are ubiquitously expressed enzymes that play essential roles in the control of many biological processes including cell growth, proliferation, apoptosis, and differentiation [1]–[3]. Considering the vast array of functions and substrates that have been attributed to PP2A family members, their activities must be tightly controlled in order to maintain cellular homeostasis. Indeed, multiple regulatory mechanisms have been reported for the phosphatase catalytic subunits (PP2Ac, PP4c, and PP6c) including a variety of post-translational modifications and their association with specific regulatory subunits. Each catalytic subunit interacts with a number of distinct canonical regulatory subunits that play a crucial role in modulating substrate selectivity and subcellular localization of the respective phosphatase holoenzyme. However, recent studies have revealed that PP2A family members also interact with atypical regulatory subunits independent of the canonical subunits. Alpha4 (α4) is one such regulatory subunit that directly binds to PP2Ac, PP4c, and PP6c [4], [5].

Alpha4, encoded by the *IGBP1* gene, is thought to be the mammalian homolog of yeast Tap42, based on their amino acid sequence similarity (24%) and the findings that both proteins interact with catalytic subunits of PP2A family members [6] (Table S1). Tap42 is an integral component of the yeast target of rapamycin (TOR) pathway. Phosphorylation of Tap42 by the nutrient-sensitive TOR kinase promotes its interaction with the yeast PP2A-like catalytic subunits Sit4 and Pph21/22, resulting in inhibition of phosphatase activities toward downstream substrates [7]. In contrast to yeast Tap42, a role for Tap42/α4 in TOR signaling in higher eukaryotes is less clear. Although some reports have implicated a role for α4 in the mammalian TOR (mTOR) pathway [8], other studies have raised questions about the involvement of α4•phosphatase complexes in this pathway [9]–[11]. In support of the idea that TOR signaling in yeast and higher eukaryotes is fundamentally different, Cygnar and colleagues demonstrated that *Drosophila* Tap42 functions independently of TOR to regulate cell division and survival [9]. α4 has also been reported to function as a key regulator of cell spreading and migration as well as an essential inhibitor of apoptosis [12], [13]. While the precise mechanism underlying Tap42/α4 regulation of phosphatase activities in higher organisms remains unclear, recent studies indicate that α4, via its interaction with the E3 ubiquitin ligase MID1, plays a crucial role in modulating PP2Ac polyubiquitination and stability [10], [14].

α4, like the PP2A-related catalytic subunits, is ubiquitously expressed in mammalian cells, and also is highly expressed in carcinogen-transformed human cells and a variety of human cancers [5], [15]. Thus, it is not too surprising that a growing number of cellular events and substrates appear to be under the control of α4 regulation of PP2A-family members. However, the *in vivo* roles of these phosphatases and α4 in specific biological processes remain unclear because knockout of these genes often leads to lethality of the organism [16], [17]. To circumvent the lethality issues, investigators have turned to conditional knockouts. While these studies have provided some insights about the function of α4 [12], [16], questions remain regarding the role of this phosphatase regulator in other biological processes, such as development.

*Drosophila* imaginal discs (primordial appendages) have proven to be a powerful experimental platform for studying poorly characterized genes and deciphering their involvement in developmental processes and specific cellular signal transduction cascades [18], [19]. The wing imaginal disc is a sac-like structure attached to the larval epidermis and composed of two epithelial layers – a columnar epithelium (disc proper, DP) and a squamous peripodial membrane (PM) or peripodial epithelium (PE) [20], [21] (Fig. S1). As the precursor of adult thorax and wings, wing discs develop internally in the larva during metamorphosis and evert, migrate, and fuse with adjacent disc derivatives [22]. During the late pupal stage, the PE degenerates but provides guidance for the patterning of DP to form the final thorax and wing structure in adults [23]. Despite the wealth of anatomical information about DP and PE, relatively little is known regarding the communication and interaction between these two epithelial layers [24], [25].

Several signal transduction pathways (e.g., JNK, DPP, and HH) are involved in the development and differentiation of the wing imaginal disc [25], [26]. The Jun-NH_2_-terminal kinase (JNK) signaling pathway is conserved from flies to humans, and plays a crucial role in stress response, apoptosis, and development [22]. The major components of the *Drosophila* JNK cascade include hemipterous (*hep*; JNKKK), slipper (*slpr*; JNKK), basket (*basket*; JNK), and DJun and DFos (*kayak*/KAY) [27]. Decapentaplegic (DPP) is the *Drosophila* homolog of the vertebrate bone morphogenetic proteins (BMPs), which are members of the TGF-β superfamily, and appears to be responsive to JNK activation [28], [29]. DPP is a morphogen that forms a concentration gradient across imaginal discs, that is essential for cell proliferation and tissue development [30]. Disruption of JNK or DPP signaling usually leads to abnormal patterning and development of the wing disc and consequential thorax and wing defects in the adult fly [29], [31]. A common phenotype seen in JNK and DPP *Drosophila* mutants is a thorax cleft, but these two pathways play different roles in the maintenance, migration, and fusion of the epithelial sheets [22]. Hedgehog (HH) signaling is also crucial for tissue development and patterning in humans as well as other organisms [26], [32]. HH binds to its receptor (Patched or Ptc) and leads to an accumulation of another receptor, Smoothened (Smo), which inhibits proteolytic cleavage of the transcription factor Cubitus interruptus (Ci) allowing Ci to diffuse into the nucleus where it induces transcription of HH target genes. In the absence of HH, Ci cleavage products (CiR) enter the nucleus and function as repressors of transcriptional activity. HH and DPP appear to direct anterior/posterior axis patterning in the developing *Drosophila* wing by functioning as short- and long-range morphogens, respectively [30], [33]. PP2A and PP4 have also been implicated in the regulation of HH signaling and appear to act in an opposing manner via their ability to target different substrates in this pathway [33]–[35].

In this study, we developed a viable/non-lethal model system for the suppression of Tap42 in imaginal discs of *Drosophila* larva. RNAi-mediated silencing of *Tap42* using the *Gal4/UAS* system and two different wing imaginal disc drivers (*pnr-* and *ap-Gal4*) resulted in complex phenotypes that included a thorax cleft, undeveloped wings, and low survival rates. We show that Tap42 is preferentially expressed in the PE cells, which provide guidance for thorax and wing development. Our biochemical and genetic data reveal alterations in JNK, DPP, and HH signaling following suppression of Tap42. The complicated phenotypes observed in the Tap42 mutant flies appear to be due to the combination of deregulated cell cycle progression, signal transduction, and apoptosis. We also demonstrate that the defects seen in the *Tap42^RNAi^* mutants are direct consequences of disrupted regulation of *Drosophila* PP2A family members (Mts, PP4, and PPV) as enforced expression of wild type Tap42, but not a phosphatase binding-defective mutant of Tap42, rescued the survivorship and phenotype of mutant flies. The experimental platform described herein provides a valuable system for investigating the *in vivo* function and regulation of Tap42•phosphatase complexes, which can be exploited to identify signaling pathways and specific substrates under the control of Tap42-regulated phosphatases.

## Results

### Phenotypes of *Drosophila* expressing tissue-specific *Tap42^RNAi^*

Depletion of the α4 and *Tap42* genes in mice and *Drosophila*, respectively, causes lethality at the early embryonic stage [9], [12], making them unsuitable for studying the *in vivo* function of α4/Tap42. To circumvent the lethality issue and to establish a model system in which the physiological consequences of *Tap42* mutants can be monitored during development, we exploited the *Drosophila Gal4/UAS* system [36], [37] for tissue-specific suppression of *Tap42*. Three wing imaginal disc-specific drivers (*pnr-Gal4*, *ap-Gal4*, and *dpp-Gal4*), a universally active driver (*actin-Gal4*), and an eye/antennae-specific driver (*GMR-Gal4*) were used to express hairpin RNAi targeting the *Tap42* gene (*UAS-Tap42^RNAi^*). Although global suppression of *Tap42* gene via the *actin-Gal4* driver caused complete lethality, no obvious abnormalities were observed in the compound eye following *Tap42* knockdown using the *GMR-Gal4* driver (data not shown). Suppression of *Tap42* in the *dpp* domain did not yield any apparent phenotype (data not shown); however, *Drosophila* expressing *Tap42^RNAi^* with the other wing imaginal disc drivers, *pnr-Gal4* and *ap-Gal4,* exhibited noticeable phenotypes and decreased survival rates.

**Figure 1:**
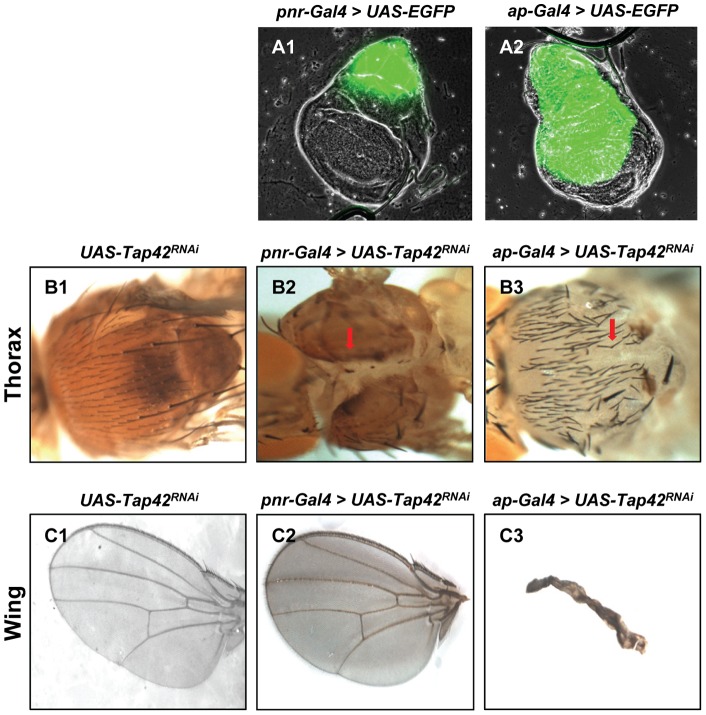
Silencing of *Tap42* in wing discs leads to pleiotrophic defects that include deformed thorax and wings. *pnr-Gal4* and *ap-Gal4* imaginal disc drivers were used to drive expression of *EGFP* or *Tap42^RNAi^* in *Drosophila*. Wing discs obtained from 3^rd^ instar larvae expressing EGFP (green) reveal the *pnr-Gal4* (A1) and *ap-Gal4* (A2) expression domain in wing discs. Control flies harboring the *UAS-Tap42^RNAi^* construct lacked any noticeable defect in the adult thorax (B1, with head left) or wing (C1, with wing margin to left). *Tap42^RNAi^* expression using the *pnr-Gal4* driver caused a marked cleft phenotype on the adult thorax (B2, red arrow) with no notable defects in fly wing (C2). Silencing the *Tap42* gene with the *ap-Gal4* driver resulted in a thorax cleft phenotype ranging in severity from mild (B3, red arrow) to severe (Fig. 6-B1) as well as drastically shriveled wings (C3). Genotypes: (A1) *UAS-EGFP/+; pnr-Gal4/+*. (A2) *ap-Gal4/UAS-EGFP*. (B1 & C1) *UAS-Tap42^RNAi^/+* as control. (B2 & B3) *UAS-Tap42^RNAi^/+; pnr-Gal4/+*. (C2 & C3) *ap-Gal4/UAS-Tap42^RNAi^; +/+*.

As revealed by EGFP expression, *pnr-Gal4* activity is restricted to the notum area of the wing disc (Fig. 1-A1), which gives rise to the adult thorax [20], [21]. Suppression of *Tap42* in the *pnr* domain resulted in the appearance of a marked thorax cleft (Fig. 1-B2), but the wings appeared normal (Fig. 1-C2). In comparison to *pnr-Gal4* activity, *ap-Gal4* activity extends from the stalk to the dorsal/ventral boundary and not only includes the notum, but hinge and wing compartments as well (Fig. 1-A2). As expected, given the broader activity of the *ap-Gal4* driver in the wing discs, *Drosophila* lines expressing *Tap42^RNAi^* under the control of *ap-Gal4* exhibited more complex phenotypes that included varying degrees of a cleft thorax (Fig. 1-B3) as well as significant wing deformities (Fig. 1-C3). Necrosis of the front leg joints was also observed in some of these flies (Fig. S2-B). These findings suggest that Tap42 is involved in wing imaginal disc morphogenesis and plays a crucial role in the patterning and differentiation of wing discs.

In addition to the morphological phenotypes, we noticed that both *pnr*-*Gal4*- and *ap-Gal4-*mediated RNAi silencing of *Tap42* caused a significant reduction in the survival rate. To evaluate the impact of *Tap42* RNAi on *Drosophila* viability, the number of *Tap42^RNAi^*-expressing progeny that survived to adults were counted and expressed as a percentage of total progeny. As shown in Table 1, the actual number of *ap-Gal4>UAS -Tap42^RNAi^* adult survivors (1.6%) were substantially lower than the theoretical number of expected adult survivors (33.3%). Silencing of *Tap42* gene via the *pnr-Gal4* driver also decreased survival (13.2% actual versus 25.0% expected). The higher lethality with the *ap-Gal4* driver, as compared to the *pnr-Gal4* driver, is most likely due to the broader expression of *Tap42^RNAi^*, which leads to expansive interruption of Tap42' s normal function. The majority of *Drosophila* death, especially in the case of the *ap-Gal4>UAS-Tap42^RNAi^* flies, appeared to occur predominantly during the pupal stage as most of these flies failed to eclose from the pupal case (Fig. S2-A).

**Table 1:** The effects of *mts^XE2258^*, *Tap42^WT^*, and *Tap42^ED^* on the viability of *Tap42^RNAi^* flie ^a, b^.

Cross	F1 Progeny	Expected ratio (%)	Actual ratio (% ± SD)	Total number (n)
Cross 1	*pnr-Gal4> UAS-Tap42^RNAi^*	25.0	13.2±4.7	485
Cross 2	*ap-Gal4> UAS-Tap42^RNAi^*	33.3	1.6±1.4	276
Cross 3	*ap-Gal4> UAS-Tap42^RNAi^, mts^XE2258^*	33.3	44.9±10.2	168
Cross 4	*pnr-Gal4> UAS-Tap42^RNAi^; MKRS*	12.5	2.6±0.6	458
	*pnr-Gal4> UAS-Tap42^RNAi^; UAS-Tap42^WT^*	12.5	16.9±1.1	
Cross 5	*pnr-Gal4> UAS-Tap42^RNAi^; MKRS*	12.5	5.7±0.8	507
	*pnr-Gal4> UAS-Tap42^RNAi^; UAS-Tap42^ED^*	12.5	5.8±2.4	
Cross 6	*mts^XE2258^ / +*	50	53.7±4.3	708
	*CyO / +*	50	46.3±4.3	

a. The actual surviving ratios of F1 progeny were quantified from the following crosses:

Cross 1: +/+; pnr-Gal4/TM3, Ser ♀ x UAS-Tap42^RNAi^/CyO; +/+ ♂.

Cross 2: ap-Gal4/CyO ♀ x UAS-Tap42^RNAi^/CyO ♂.

Cross 3: ap-Gal4/CyO ♀ x UAS-Tap42^RNAi^, mts^XE2258^/CyO ♂.

Cross 4: +/+; pnr-Gal4/TM3, Ser ♀ x UAS-Tap42^RNAi^/CyO; UAS-Tap42^WT^/MKRS ♂.

Cross 5: +/+; pnr-Gal4/TM3, Ser ♀ x UAS-Tap42^RNAi^/CyO; UAS-Tap42^ED^/MKRS ♂.

Cross 6: +/+ ♀ x mts^XE2258^/CyO ♂.

b. Crosses were repeated at least three times and flies that enter eclosion were counted as survivors.

### Tap42 expression in wing imaginal discs

**Figure 2:**
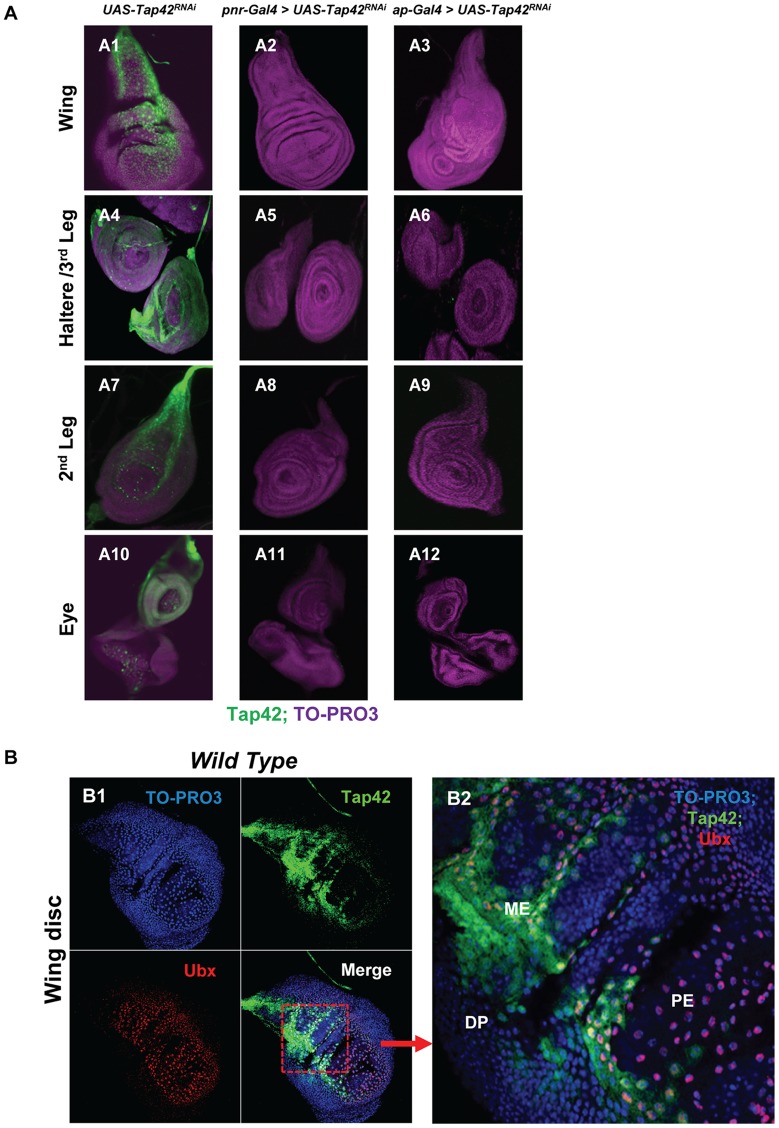
Tap42 is expressed in imaginal discs and primarily localized in the peripodial epithelium (PE) region. Panel A: Wing (A1–A3), haltere/3^rd^ leg (A4–A6), 2^nd^ leg (A7–A9), and eye imaginal discs (A10–A12) isolated from 3^rd^ instar larvae were immunostained for Tap42 protein expression (green) and counter-stained with the nucleic acid dye TO-PRO3 (purple). *UAS-Tap42^RNAi^* control flies exhibited abundant expression of *Tap42* in the PE region of these imaginal discs (A1, A4, A7, & A10). *Tap42^RNAi^* expression with the *pnr* (A2, A5, A8, & A11) and *ap* (A3, A6, A9, & A12) drivers dramatically reduced *Tap42* expression to nearly undetectable levels. Of note, *ap-Gal4*-mediated silencing of *Tap42* also disrupted the morphological patterning of the wing disc, as revealed by TO-PRO3 staining (A3). Panel B: The localization of Tap42 in the PE region was confirmed by immunofluorescence histochemistry. Immunostaining of wing discs obtained from wild type flies revealed an overlap of Ubx (red) and Tap42 (green) expression (B1). An amplified view of the merged image highlights strong Tap42 expression around the presumptive medial edge (ME) cells of the PE, which localizes near the boundary of the PE and DP (B2). Some Tap42 expression was visualized in the disc proper (DP) cells. Wing discs were counter-stained with the nucleic acid dye TO-PRO3 (blue). Genotypes: (A1, A4, A7, & A10) *UAS-Tap42^RNAi^/+* as control. (A2, A5, A8, & A11) *UAS-Tap42^RNAi^/+; pnr-Gal4/+*. (A3, A6, A9, & A12) *ap-Gal4/UAS-Tap42^RNAi^; +/+*. (B1 & B2) wild type *w^1118^*.

To begin to explore the mechanism underlying Tap42 regulation of wing disc development, we examined the expression pattern of *Tap42* in wing discs using immunofluorescence histochemistry and a Tap42-specific rabbit polyclonal antibody. Tap42 is highly expressed in the wing disc stalk and squamous peripodial epithelium (PE) cells but weakly expressed in the columnar disc proper (DP) cells (Fig. 2-A1 & Fig. S1). Silencing of the *Tap42* gene using the *pnr-* or *ap-Gal4* drivers almost completely eliminated the Tap42 signal (Fig. 2-A2 & A3), thus verifying the specificity of the Tap42 antibody and demonstrating the high efficacy of the *Tap42*-targeted RNAi. Although *pnr-Gal4* activity was found in a more restricted compartment of the wing disc as compared to *ap-Gal4* activity (Fig. 1-A1 & A2), both drivers effectively eliminated *Tap42* expression in wing disc. Interestingly, we also observed that the morphological structures and patterns of the *ap-Gal4>Tap42^RNAi^* wing disc (as revealed using the nucleus stain TO-PRO3) were disrupted in the DP cells (Fig. 2-A3), which eventually gives rise to the thorax and wings [20], [21]. However, no obvious alterations of the wing disc morphological structures and patterns were found in the *pnr-Gal4>UAS-Tap42^RNAi^* flies (Fig. 2-A2).

To determine if Tap42 expression is restricted to cells of the PE, we co-stained wing discs with antibodies recognizing Tap42 and Ubx, a marker for PE cells [20]. As shown in Fig. 2-B1 and B2, the Tap42 immunostaining partially overlapped the Ubx-positive cells. Particularly strong Tap42 expression was seen in the presumptive “medial edge cells” along the boundary of the PE and DP, which are thought to be involved in the formation of dorsal midline during metamorphosis [22] (Fig. S1). Although Tap42 is broadly expressed in the PE, we observed distinct subpopulations of cells in the PE that lack Tap42. We also observed a number of columnar DP cells staining positive for Tap42. Therefore, Tap42 may act as a potential marker to monitor the developmental fate and roles of a distinct subgroup of cells in imaginal discs during *Drosophila* development.

### Distribution of Tap42 in other imaginal discs and tissues

The imaginal discs attach to the larvae epidermis via a stalk and differentiate into a variety of adult cuticles. Although their developmental fates differ, imaginal discs share some structural similarities and contain both peripodial epithelial and disc proper epithelial layers [19], [21]. Therefore, we examined the distribution of Tap42 in other imaginal discs and found that it is abundantly expressed in haltere, leg, and eye-antenna discs in a pattern reminiscent of that seen in the wing discs (Fig. 2-A4, A7 & A10). The Tap42 signal was predominantly localized to the stalk and peripodial membrane in the haltere and 3^rd^ leg (Fig. 2-A4), and 2^nd^ leg (Fig. 2-A7). In the eye-antenna disc, extensive Tap42 expression was observed in the stalk and the posterior half of the central knob in the upper half of the disc (Fig. 2-A10), which gives rise to the adult antenna. We also noticed some Tap42 signal in a region containing photoreceptor cells. Since the mammalian homolog of Tap42, α4, is expressed in diverse tissue types including brain, muscle, and intestine [38], we also examined the expression profile of Tap42 in several different tissues of the adult *Drosophila*. Consistent with the ubiquitous expression profile of α4, we detected Tap42 in neurons, brain, and gut (data not shown). The absence of a noticeable defect in the adult eye suggests that Tap42 may differentially regulate the development and signaling of various tissues.

Although *pnr-Gal4* and *ap-Gal4* have frequently been classified as wing disc-specific drivers, recent studies indicate that these two genes appear to be expressed in multiple imaginal discs and tissues [39], [40]. In line with these studies, we found that *ap* and *pnr* activities were not restricted to wing discs as *pnr-Gal4* and a*p-Gal4* mediated RNAi silencing of *Tap42* also eliminated its expression in the other discs (Fig. 2-A5, A8, A11 & A6, A9, A12). However, major external morphological defects could only be detected in the adult thorax and wing (Fig. 2-A1), thus suggesting that Tap42 plays a crucial role in the development of the wing disc but relatively minor roles in other discs such as the eye, haltere, and leg discs.

### RNAi-mediated silencing of Tap42 impacts multiple signaling pathways

**Figure 3:**
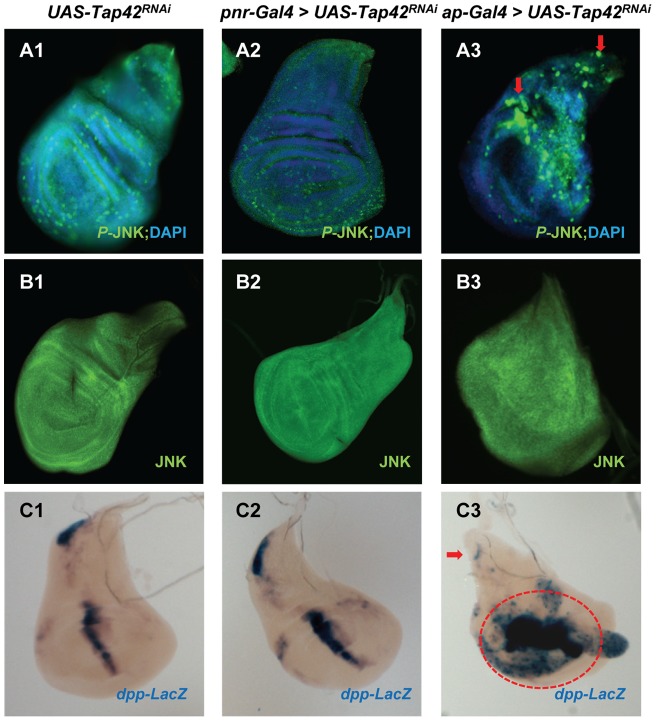
JNK and DPP signaling are altered in wing imaginal discs following depletion of Tap42. The activity and expression of BSK was monitored in wing imaginal discs using antibodies recognizing phospho-JNK or total JNK. The pattern of active JNK/BSK (green, A1-3) was not different between control *UAS-Tap42^RNAi^* flies (A1) and flies co-expressing the *pnr* driver (A2). However, hyperphosphorylation of JNK/BSK was observed in the wing disc dorsal compartment (red arrows) along with hypophosphorylation of JNK/BSK in the ventral wing compartment when *Tap42^RNAi^* was driven by *ap-Gal4* (A3). Total levels of JNK/BSK (green, B1-B3) did not change as a result of *Tap42* knockdown. *Dpp* gene expression (purple, C1-C3), as monitored by X-GAL staining of *dpp-LaZ*, in the scutellum and along the anterior/posterior boundary of the wing blade was similar in both control (C1) and *pnr*-*Gal4* driven *Tap42^RNAi^* flies (C2). *ap-Gal4* driven *Tap42^RNAi^* flies demonstrated decreased DPP signal in the scutellum (red arrow, C3) and expanded staining in the wing blade compartment (red dashed line, C3). Genotypes: (A1, B1, & C1) *UAS-Tap42^RNAi^/+* as control. (A2, B2, & C2) *UAS-Tap42^RNAi^/+; pnr-Gal4/+*. (A3, B3, & C3) *ap-Gal4/UAS-Tap42^RNAi^; +/+*.

To explore the molecular mechanism underlying the thorax and wing phenotypes of *Tap42^RNAi^* flies, we examined a number of signaling pathways that are known to be involved in the control of wing disc development. We initially monitored JNK and DPP signaling as these pathways play important roles in the epithelium sheet migration and fusion, and their disruption can lead to a remarkable thorax cleft phenotype [22], [28]. The activity of *Drosophila* JNK (BSK) was assessed by immunostaining the discs with a phospho-specific antibody recognizing the active form of JNK. Suppression of the *Tap42* gene in the *pnr* gene domain did not have a significant effect on the p-JNK signal in the scutellum area of the dorsal compartment (Fig. 3-A2), which develops into the adult notum. However, silencing of *Tap42* in the *ap* gene domain had a profound effect on the JNK activity pattern in the wing discs, especially along the ventral/dorsal boundary, as evident by hyperphosphorylation of JNK in the dorsal side and almost complete loss of p-JNK in the ventral part (Fig. 3-A3 & Fig. S1). Overexpression of a dominant-negative BSK in the *ap* domain failed to rescue the *Tap42^RNAi^* thorax cleft phenotype (Fig. S3-A & B). Together, these findings indicate that alterations in JNK signaling contribute very little, if any, to formation of the thorax cleft in *Tap42^RNAi^* flies.

We utilized the *dpp-LacZ* reporter to determine whether DPP expression was altered in *Tap42^RNAi^*-expressing wing discs. X-GAL staining revealed that *ap-Gal4-*mediated RNAi silencing of *Tap42* increased *dpp* gene expression around the wing blade but effectively eliminated its expression in the scutellum area, as compared with control flies (Fig. 3-C3). In contrast to *ap-Gal4>UAS-Tap42^RNAi^* wing discs, we did not detect any significant changes in DPP expression in wing discs expressing *Tap42^RNAi^* under the control of *pnr-Gal4* (Fig. 3-C2). Thus, while the loss of DPP expression in the scutellum of *ap-Gal4>UAS-Tap42^RNAi^* flies may contribute to the thorax cleft phenotype, the lack of any significant alterations in DPP expression in *pnr-Gal4>UAS-Tap42^RNAi^* wing discs indicate that DPP levels are probably not solely responsible for formation of the thorax cleft in *Tap42^RNAi^* flies.

**Figure 4:**
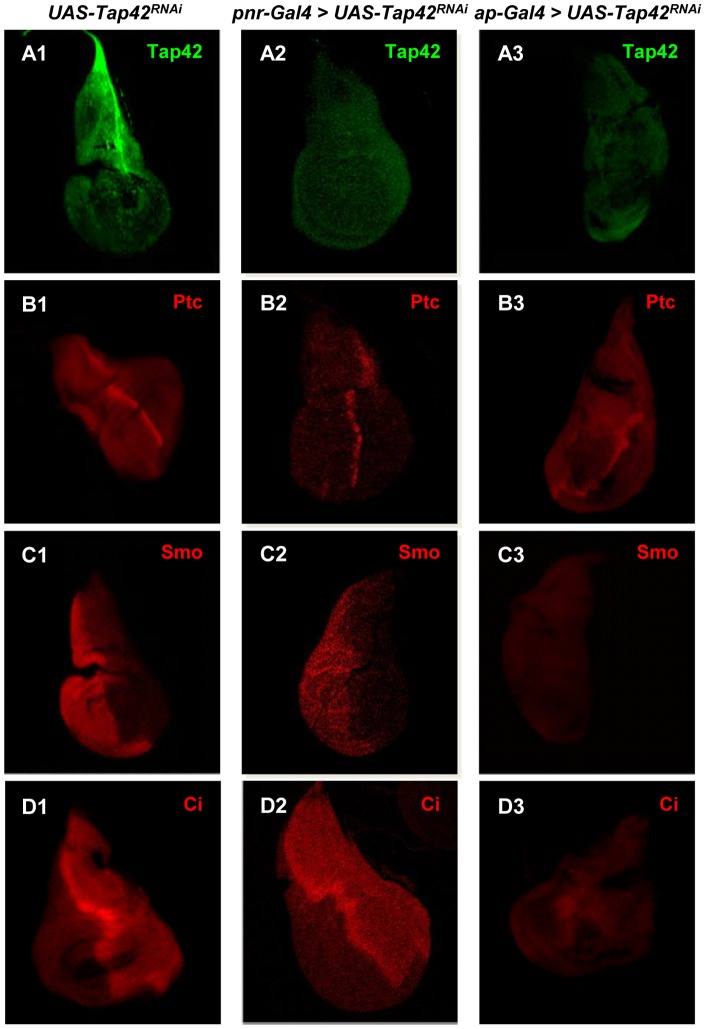
Suppression of Tap42 expression in wing imaginal discs interrupts HH signaling, hampers mitosis, and triggers apoptosis. Panel A: Isolated wing imaginal discs were immunostained with antibodies recognizing Tap42 (green) and multiple components in the HH signaling pathway, including Ptc, Smo, and Ci (red). Control wing discs displayed strong Tap42 (A1) expression and the expected expression pattern for Ptc (B1), Smo (C1), and Ci (D1). Suppression of *Tap42* with the *pnr-Gal4* or ap*-Gal4* driver effectively reduced Tap42 levels in wing discs (A2 & A3). While the levels of the HH receptor Ptc were unaffected by Tap42 silencing (B3), the expression of other downstream components of HH signaling, Smo (C3) and Ci (D3), were abrogated. Suppression of Tap42 with the *pnr-Gal4* driver did not alter the expression pattern of HH signaling as shown in B2 (Ptc), C2 (Smo) and D2 (Ci). Genotypes: (A1, B1, C1, & D1) *UAS-Tap42^RNAi^/+* as control. (A2, B2, C2, & D2) *UAS-Tap42^RNAi^/+; pnr-Gal4/+*. (A3, B3, C3, & D3) *ap-Gal4/UAS-Tap42^RNAi^; +/+*.

The morphological changes seen in the *ap-Gal4>UAS-Tap42^RNAi^* wing discs could also be due to alterations in the HH signaling pathway, which has been shown to modulate DPP activity and plays a crucial role in regulation and patterning of the discs during development [28]. Given that both PP2A/Mts and PP4 have been implicated in the control of HH signaling and wing development [33]-[35], we examined the effects of *Tap42^RNAi^* on various components of this pathway. Silencing of *Tap42* using the *ap-Gal4* driver did not have any noticeable effects on the levels or expression pattern of Ptc (HH receptor) (Fig. 4-B3), but led to suppressed expression of the downstream effectors of HH signaling, Smoothened (Smo) and Cubitus interruptus (Ci) (Fig. 4-C3 & D3). In contrast to the *ap-Gal4>UAS-Tap42^RNAi^* wing discs, silencing of *Tap42* in *pnr* gene domain did not alter the expression pattern of HH components (Fig. 4-B2, C2, & D2). Our cumulative analyses of *ap-Gal4>UAS-Tap42^RNAi^* wing discs indicate that Tap42's modulation of HH, DPP, and JNK signaling is required for normal wing imaginal disc development.

### Silencing of Tap42 hampers mitosis and triggers strong apoptosis

**Figure 5:**
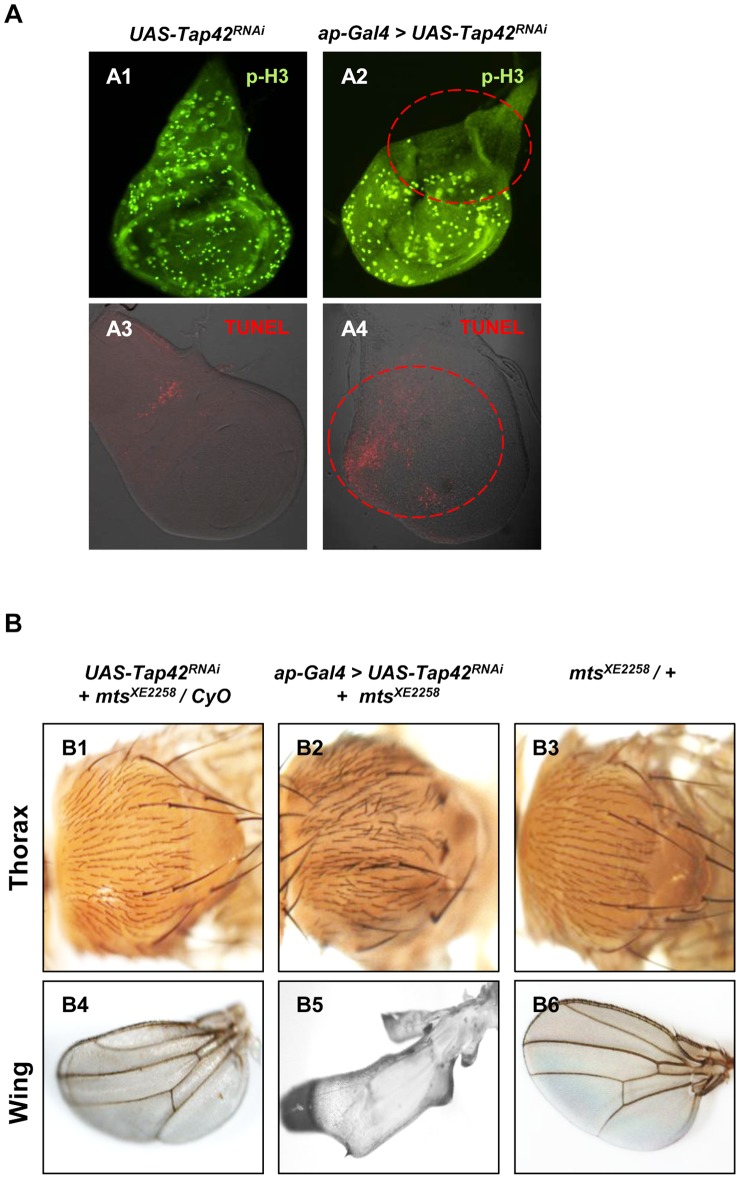
The *mts^XE2258^* allele partially rescues *Tap42^RNAi^*-induced thorax and wing phenotypes. Panel A: Mitosis and apoptosis in wing discs were monitored using a phospho-Histone H3 (p-H3, green) antibody and TUNEL staining (red), respectively. Control wing discs exhibited phospho-Histone expression throughout the wing disc (A1) with sporadic apoptotic signals (A3). *Tap42^RNAi^* under the control of the *ap* driver arrested mitosis in the notum area (red dashed line, A2) and triggered massive apoptosis, especially within the wing compartment (red dashed line, A4). Genotypes: (A1 & A3) *UAS-Tap42^RNAi^/+* as control. (A2 & A4) *ap-Gal4/UAS-Tap42^RNAi^; +/+*. Panel B: Adult control flies (*UAS-Tap42^RNAi^; mts^XE2258^*), as well as flies harboring the *mts^XE2258^* allele alone, did not exhibit any noticeable defect in the thorax (B1 & B3) or wings (B4 & B6). Introduction of the *mts^XE2258^* allele into the *Tap42^RNAi^* background resulted in a milder thorax cleft phenotype as compared to flies lacking the *mts^XE2258^* allele (compare B2 with Figs. 1-B3 & 6-B1). Furthermore, the presence of the *mts^XE2258^* allele resulted in a more developed wing (compare B5 with Fig. 1-C3). Genotypes: (B1 & B4) *UAS-Tap42^RNAi^, mts^XE2258^/CyO*. (B2 & B5) *ap-Gal4/UAS-Tap42^RNAi^, mts^XE2258^.* (B3 & B6) *mts^XE2258^*/+.

Since PP2A family members have been implicated in the regulation of cell proliferation and mitosis [1], [9], we asked whether suppression of their common regulatory subunit, Tap42, in wing discs influences these cellular processes. Proliferating cells undergoing mitosis were visualized using a phospho-histone3 antibody, which is a marker of cells in late G_2_ and M phase [9]. As shown in Fig. 5-A2, cell proliferation was arrested within the notum region of wing discs harboring *Tap42^RNAi^* under the control of the *ap* driver, but no obvious changes in cell proliferation were observed in the wing compartment. TUNEL staining also revealed strong apoptosis around the wing blade in *ap-Gal4>Tap42^RNAi^* wing discs but only random apoptotic signals were found in the control discs (Fig. 5-A4). Although it remains to be determined whether defective cell cycle progression and apoptosis are direct consequences of *Tap42* knockdown, alterations in these biological processes could provide an explanation for the morphological defects seen in the *ap-Gal4>Tap42^RNAi^* wing discs and the adults.

### The *mts^XE2258^* allele partially relieves the Tap42 RNAi-induced phenotypes

We next asked whether the *Tap42^RNAi^*-induced phenotypes are influenced following introduction of a heterozygous mutant of the PP2A catalytic subunit, *mts^XE2258^*, which displays reduced phosphatase activity [41]. This allele itself did not display any noticeable phenotype in thorax and wings (Fig. 5-B3 & B6), nor any significant impact on fly survival rate (Cross 6 in Table 1). However, introduction of the *mts^XE2258^* allele into flies expressing *Tap42^RNAi^* within the *ap* gene domain (*ap-Gal4>UAS-Tap42^RNAi^; mts^XE2258^*) caused a significant rescue of the cleft thorax when compared with flies expressing *Tap42^RNAi^* alone (compare Figs. 1–B3 & 5-B2). The double mutant also exhibited blistered, albeit more developed wings, as compared to the totally shriveled wings seen in the *ap-Gal4>UAS-Tap42^RNAi^* flies (compare Figs. 1-C3 & 5-B5). We also compared the survivor rates of *ap-Gal4>UAS-Tap42^RNAi^; mts^XE2258^,* and ap*-Gal4>UAS-Tap42^RNAi^* flies. *ap-Gal4*-mediated expression of *Tap42^RNAi^* in the *mts^XE2258^* background had a profound effect on *Drosophila* survival rates, increasing the survivor/total progeny ratio from 1.6% (*ap-Gal4>UAS-Tap42^RNAi^*) to 44.9% (*ap-Gal4>UAS-Tap42^RNAi^; mts^XE2258^*) (Cross 2 & 3 in Table 1), thus indicating that the *mts^XE2258^* allele abrogates the lethal effect generated by suppression of Tap42 gene in *ap* gene domain. These findings demonstrate that Tap42's modulation of Mts plays an active role in *Drosophila* tissue development and viability.

### Tap42 interacts with all three *Drosophila* PP2A family members (Mts, PP4, and PPV)

**Figure 6:**
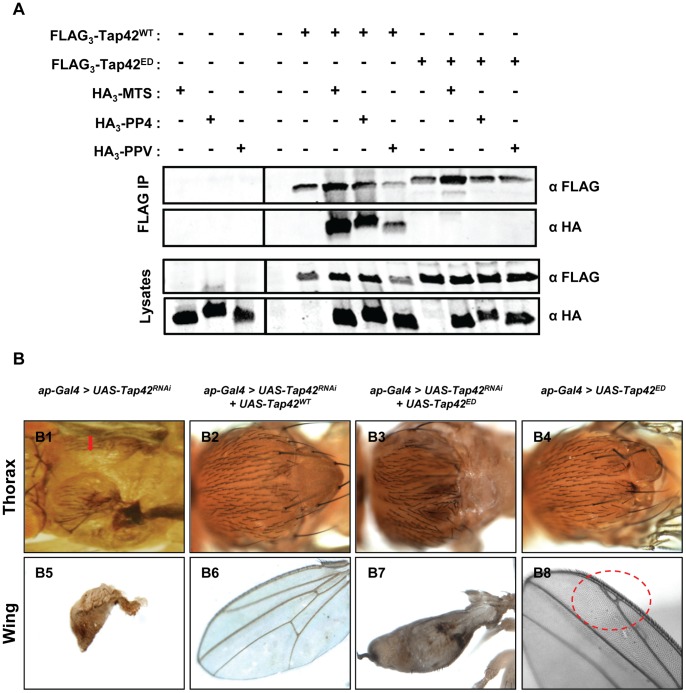
Tap42 interacts with all three PP2A members and is required for normal wing disc development. Panel A: FLAG immunoprecipitations (FLAG IPs) were performed from extracts of *Drosophila* S2 cells expressing HA_3_-Mts, HA_3_-PP4, or HA_3_-PPV alone or together with wildtype (FLAG_3_-Tap42^WT^) or mutant Tap42 (FLAG_3_-Tap42^ED^). The FLAG immune complexes and corresponding cell extracts (lysates) were analyzed by Western blotting using the indicated epitope tag antibodies. Panel B: Adult flies expressing *Tap42^RNAi^* in the *ap* domain displayed a marked thorax cleft (red arrow, B1) and shriveled wings (B5). Expression of *Tap42^WT^* in this background completely rescued both thorax (B2) and wing defects (B6). However, introduction of the *Tap42^ED^* mutant in this background failed to rescue the defects and the flies lacked the scutum (B3) and formed blistered wings (B7). Expression of *Tap42^ED^* alone resulted in a mild defect around the scutum (B4) and the formation of a forked wing vein (B8). Genotypes: (B1 & B5) *ap-Gal4/UAS-Tap42^RNAi^; +/+*. (B2 & B6) *ap-Gal4/UAS-Tap42^RNAi^; +/UAS-Tap42^WT^*. (B3 & B7) *ap-Gal4/UAS-Tap42^RNAi^; +/UAS-Tap42^ED^.* (A4 & B4) *ap-Gal4/+; +/UAS-Tap42^ED^*.

Our analysis of *ap-Gal4>UAS-Tap42^RNAi^; mts^XE2258^* flies implicates a crucial role for Tap42 and Mts in normal fly development; however, *Drosophila* PP4 and PP6 (PPV) may also be involved in this process as the mammalian homolog of Tap42, α4, interacts with all three PP2A family members [4], [5]. To test if Tap42 interacts with Mts, PP4, and PPV, we performed FLAG immunoprecipitations from lysates of *Drosophila* S2 cells expressing the HA_3_-tagged phosphatase alone or together with FLAG_3_-Tap42^WT^. Western analysis of the immune complexes revealed that Tap42 interacts with all three *Drosophila* phosphatase catalytic subunits (Fig. 6-A). Since prior studies have identified a double point mutant of α4 that lacks the PP2Ac binding determinants [10], [42], we mutated the corresponding residues in Tap42, R152E152 and K155D155, and monitored the ability of this mutant (Tap42^ED^) to interact with Mts, PP4, and PPV. In contrast to wild type Tap42 (Tap42^WT^), Tap42^ED^ failed to interact with the *Drosophila* phosphatases (Fig. 6-A).

### The *Tap42^RNAi^*-induced phenotypes are strictly dependent on Tap42's interaction with PP2A family members

To test if phosphatase binding contributes to the *Tap42^RNAi^*-induced phenotypes, we expressed *UAS-Tap42^WT^* and *UAS-Tap42^ED^* in flies and monitored their effects on survival rates and tissue development. We first examined the effects of *Tap42^WT^* and *Tap42^ED^* overexpression alone in the *ap* domain of wing discs. Although no obvious defects were observed in the thorax and wing of *ap-Gal4>UAS-Tap42^WT^* flies (not shown), overexpression of *Tap42^ED^* resulted in a smaller notum lacking the scutum (Fig. 6-B4) and forked veins on the wings (Fig. 6-B8). These findings indicate that the phosphatase binding-defective mutant of Tap42, which mildly disrupts the developmental process, may function as a dominant-negative in the control of thorax development.

We next introduced both *UAS-Tap42^WT^* and *UAS-Tap42^ED^* into the *Tap42^RNAi^* backgrounds, and monitored the phenotypical consequences of these genetic manipulations. Flies co-expressing *Tap42^WT^* and *Tap42^RNAi^* under the control of the *ap-Gal4* driver displayed normal development of thorax and wings (Fig. 6-B2 & B6), thus validating the specificity of RNAi and demonstrating that the expression of the wild type protein reverts the *Tap42^RNAi^* phenotypes. In contrast to wild type *Tap42*, overexpression of the phosphatase binding-defective mutant (*Tap42^ED^*) failed to rescue the *Tap42^RNAi^*-induced phenotypes (e.g., thorax cleft and wing deformities) (Fig. 6-B3 & B7). A similar rescue was observed when co-expression was driven by *pnr-Gal4* (Fig. S4).

We also examined the effects of *Tap42^WT^* and *Tap42^ED^* overexpression on the viability of *Tap42^RNAi^* flies. For these studies, we utilized the *pnr* driver as the genetic manipulations were more feasible. The survival rate of *Tap42^RNAi^* flies (2.6%) increased substantially following introduction of wild type Tap42 (16.9%) (compare Cross 4 & 5 in Table 1). However, expression of *Tap42^ED^* failed to improve the survival rate, and the number of survivors was comparable to that of flies expressing *Tap42^RNAi^* alone. Together, these findings establish a crucial role for Tap42 modulation of PP2A family members in the control of *Drosophila* development and viability.

## Discussion

Our understanding about the *in vivo* function of α4/Tap42, especially in development, is limited in part because global knockout of this gene in mice and flies leads to early embryonic death [9], [12]. Cellular studies have also revealed that depletion of α4/Tap42 causes death in embryonic stem cells, mouse embryonic fibroblasts, adipocytes, hepatocytes, B and T cells of the spleen and thymus, and *Drosophila* S2 cells [11], [12], [16]. Although studies of a conditional (*Cre-LoxP*) α4 knockout in mouse hepatocytes and a mosaic assay of Tap42 in *Drosophila* wing disc have provided insights into the cellular biology of α4 and Tap42 [9], [12], the impact of these gene products on the development of tissues and host have not yet been described. In this report, we utilized *Tap42*-targeted RNAi and the *Gal4/UAS* system to investigate the biological effects of silencing *Tap42* expression in specific *Drosophila* tissues. Suppressing the *Tap42* gene using two tissue-specific drivers (*pnr-Gal4* and *ap-Gal4*) led to a pleiotropic fly phenotype, which included major deformities in the adult thorax and wings as well as decreased survival rates. The experimental platform described herein has allowed us to explore the role of Tap42 and Tap42-regulated phosphatases in the control of cellular signaling, tissue development, and *Drosophila* viability.

Our analyses of *Tap42^RNAi^* wing discs revealed significant alterations in multiple signal transduction pathways including JNK, DPP, and HH. Marked increases in p-JNK signals were found in *ap-Gal4>Tap42^RNAi^* wing discs (Fig. 3-A3). This observation, together with previous studies showing increased c-Jun phosphorylation in α4-null mouse embryonic fibroblasts [12] and activated JNK in Tap42-depleted clones of fly wing discs [9], indicate that α4/Tap42 likely plays a negative role in regulation of JNK signaling. Silencing the *Tap42* gene in the *ap* gene domain also changed DPP and HH signaling in the wing discs (Figs. 3-C3, 4-C3, & 4-D3). Although *ap-Gal4*-mediated silencing of *Tap42* had a profound effect on JNK, DPP, and HH signaling, these pathways were unaffected in *pnr-Gal4>Tap42^RNAi^* wing discs (Fig. 3-A2 and C2), thus demonstrating that the thorax cleft phenotype seen in the *pnr-Gal4>Tap42^RNAi^* flies is not due to alterations in these pathways. Collectively, these findings indicate that Tap42 plays a crucial role in the modulation of JNK, DPP, and HH signaling, but the effects of Tap42 on these pathways appear to play a minimal role in normal thorax development.

The HH pathway is one of the major guiding signals for imaginal disc development [26], [30]. Recent investigations have revealed that the phosphorylation state of Ci and Smo, two components of the HH signaling pathway, are controlled by *Drosophila* PP2A (Mts) and PP4 [33]. Additional studies implicate a role for specific Mts complexes in the control of HH signaling, whereby holoenzyme forms of Mts containing the Wdb and Tws regulatory B subunits act at the level of Smo and Ci, respectively [35]. Together, these findings point to key roles for Mts and PP4 in HH signaling and suggest that a common subunit of these phosphatases, namely Tap42, may also be involved in HH signaling. Indeed, our data clearly show that Tap42 plays an important regulatory role in this pathway as silencing of Tap42 within the wing discs leads to an elimination of both Smo and Ci expression (Fig. 4-C3 & D3). Although the precise role(s) of Tap42 in the control of HH signaling remains unclear, it likely involves Tap42-dependent regulation of one or more phosphatase catalytic subunits (e.g., Mts, PP4, and possibly PPV) or specific holoenzymes forms of these phosphatases (e.g., Wdb/Mts, Tws/Mts). The pleiotropic effects of *Tap42^RNAi^* on JNK, DPP, and HH signaling could be due to loss of Tap42's regulation of phosphatase activity, cellular levels, holoenzyme assembly, or subcellular localization.

Depletion of α4 in mouse embryonic fibroblasts caused an increase in phosphorylation of a variety of established PP2A substrates, which was attributed to a “generalized defect in PP2A activity” [43]. Instead of the expected unidirectional increase in protein phosphorylation, our findings demonstrate a dual role for Tap42 in the control of JNK activation as hyperphosphorylation and hypophosphorylation of JNK were observed in the dorsal and ventral sides of the *Tap42^RNAi^* wing disc, respectively, relative to control wing discs (Fig. 3-A3). Silencing of *Tap42* in the *ap* domain also impacted DPP in a bi-directional fashion; these flies exhibited significantly decreased DPP expression in the scutellum but augmented expression around the wing blade (Fig. 3-C1 & C3). Consistent with previous studies showing that PP2A functions at different levels within the Ras1 and HH pathways [35], [47], our data indicate that Tap42-regulated phosphatases likely target multiple substrates within the JNK and DPP pathways in different regions of wing discs.

Close examination of the PE cells in the wing disc revealed that *Tap42* expression occurs in only a fraction of these cells (Fig. 2-B1). It is noteworthy that the majority of Tap42 localized in rows of cells delineating the PE/DP boundary (Fig. 2-B2). These cells are commonly referred to as “medial edge” cells [22], [44], which represent a subpopulation of PE cells that play a crucial role in thorax closure during metamorphosis [21], [22], [31], [45]. Interestingly, α4•PP2A complexes appear to play a major role in the control of cell spreading, migration, and cytoskeletal architecture, presumably via their ability to modulate the activity of the small G-protein Rac [13]. Yeast Tap42 has also been implicated in the cell cycle-dependent and polarized distribution of actin via a Rho GTPase-dependent mechanism [46]. Therefore, we hypothesize that the wing disc structural deformities and thorax cleft phenotype of *Tap42^RNAi^* flies are a result of unregulated phosphatases leading to defective spreading and migration of the medial edge cells during metamorphosis. The thorax cleft phenotype provides an opportunity to delineate the precise roles of Tap42•phosphatase complexes in processes controlling thoracic closure (e.g., cell spreading and migration).

α4/Tap42 appears to function as an essential anti-apoptotic factor as cells lacking this common regulatory subunit of PP2A family members undergo rapid death [9], [12]. These studies implicate a role for α4/Tap42-dependent regulation of PP2A-like enzymes, and presumably the phosphorylation state of multiple pro- and anti-apoptotic proteins, in the maintenance of cell survival. Our findings reveal that silencing Tap42 in wing discs triggers apoptosis (Fig. 5-A4), thus providing supportive *in vivo* evidence that depletion of Tap42 (α4) leads to deregulated phosphatase action, which switches these enzymes from pro-survival to pro-apoptotic mediators. Because JNK activation is a hallmark feature of apoptosis [47], the overlap of apoptotic cells and hyperphosphorylated JNK (compare Fig. 3-A3 & Fig. 5-A4) indicates that the *Tap42^RNAi^*-induced apoptosis may be dependent on JNK activation.

Since α4 is required for maintaining the normal function of PP2A, PP4, and PP6 [12], we suspected that misregulation of these phosphatases could be responsible for the pleiotrophic phenotypes observed in *Tap42^RNAi^* flies. Consistent with this idea, introduction of the *mts^XE2258^* heterozygous allele into *ap-Gal4>UAS-Tap42^RNAi^* flies partially rescued the thorax and wing defects (Fig. 5-B2 & B5), and significantly improved fly survival rates (compare Cross 2 & 3 in Table 1). The partial rescue by *mts^XE2258^* suggests that the defects seen in the *Tap42^RNAi^* flies are due, in part, to unregulated Mts activity, possibly as a result of increased Mts levels or enzymatic activity. Indeed, previous studies have demonstrated an accumulation of Mts in *Tap42*-depleted clones of the fly wing disc [9]. Thus, *mts^XE2258^* appears to function as a mild mutant that partially restores misregulated Mts function following depletion of *Tap42*. However, given our biochemical findings showing that Tap42 also interacts with PP4 and PPV (Fig. 6-A), additional studies will be needed to determine the relative contribution of these phosphatases to the *Tap42^RNAi^*-induced defects.

The phenotypes observed in flies expressing *Tap42^RNAi^* could also be attributed to loss of a phosphatase-independent function(s) of Tap42 that controls normal fly development. However, introduction of a phosphatase binding-defective mutant of Tap42 (*Tap42^ED^*) into the *Tap42^RNAi^* background failed to rescue the phenotypes and lethality associated with Tap42 depletion (Fig. 6-B). In contrast to *Tap42^ED^*, introduction of *Tap42^WT^* fully rescued the phenotypes and lethality of the *Tap42^RNAi^* flies. These findings indicate that the *Tap42^RNAi^*-induced phenotypes are entirely due to the impaired interactions between Tap42 and PP2A family members, and provide compelling support for the hypothesis that Tap42-dependent regulation of the functions of these enzymes is crucial for normal wing disc development and *Drosophila* viability.

Although we are still far from understanding the exact molecular mechanisms underlying Tap42's regulation of PP2A family members, our studies clearly demonstrate that Tap42-phosphatase interactions play crucial roles in the control of multiple signaling pathways governing cell growth and survival. The experimental platform described in this report will undoubtedly serve as a valuable system to further explore the *in vivo* function and regulation of Tap42•phosphatase complexes. Furthermore, given the remarkable phenotypes seen in the *Tap42^RNAi^* flies (e.g., thorax cleft and deformed wings), we anticipate that this model system will drive future studies (e.g., phenotype-based suppressor/enhancer screens) aimed at identifying direct targets of Tap42-regulated phosphatases, as well as additional pathways under the control of these phosphatase complexes.

## Materials and Methods

### Plasmids

The full-length *Tap42* cDNA was amplified by PCR from the DGRC clone (LD07294) and inserted into the pENTR/D-TOPO vector (Invitrogen, Carlsbad, CA). Expression plasmids were generated by swapping the pENTR-*Tap42* entry vector into destiny vectors containing different epitope tags, such as *pAct5C-FLAG-Tap42^WT^ (wild type), pAct5C-3HA-mts, pAct5C-3HA-PP4, pAct5C-3HA-PPV.* The *pAct5C-FLAG-Tap42^ED^ and pUAS-Tap42^ED^* plasmids, which harbor R152E and K155D mutations, were generated using the Quick Change ®II Site-Directed Mutagenesis Kit (Agilent Technologies, Palo Alto, CA) and the pENTR-*Tap42^WT^* vector as a template, and then swapping the construct into the corresponding destiny vectors.

### Drosophila stocks

The *Tap42^RNAi^* (GD27179) *Drosophila* strain was obtained from the Vienna Drosophila RNAi Center (VDRC). Fly strains *mts^XE2258^*, *dpp-Gal4* (#1553), *pnr-Gal4* (#3039) [48], *ap-Gal4* (#3041) [39], *actin5C-Gal4* (#3954), *UAS-2xEGFP* (#6874), *GMR-Gal4* (#8121), *dpp-lacZ* (#8412) [22], and 2^nd^ chromosome balancer CyO with *actin-GFP* transgene (#4533) were obtained from the Bloomington Drosophila Stock Center (BDSC). The *UAS-Tap42^WT^* (wild type) fly was a generous gift from Dr. Thomas Neufeld and described previously [9]. Other fly strains and chromosomes are as described in the Flybase. Transgenic flies harboring *UAS-Tap42^ED^* were generated by injection of pUAS-*Tap42* ^ED^ vector using a standard protocol. All fly strains were kept at room temperature with 12 h light/dark cycles and subject to standard genetic cross protocols.

### Antibodies

The HA and FLAG mouse monoclonal antibodies were obtained from Roche (Indianapolis, IN) and Sigma-Aldrich (St. Louis, MO), respectively. The p-JNK rabbit antibody and the JNK rabbit antibodies were from Promega (Madison, WI) and Santa Cruz Biotechnology (Santa Cruz, CA), respectively. The Ptc, Smo, and Ci antibodies were obtained from the Developmental Studies Hybridoma Bank (DSHB, University of Iowa). The AlexFluor488-conjugated goat anti-rabbit and AlexFluor568-conjugated goat anti-mouse antibodies were obtained from Invitrogen (Carlsbad, CA). GST-Tap42 purified from E. *coli* was used as an immunogen for rabbit antibody production (Bethyl Laboratories, Montgomery, TX), and antibodies were purified from the sera using Protein A Sepharose 4B matrix (Invitrogen, Carlsbad, CA) [49].

### S2 cell culture and transfection

S2 cells were maintained at 25°C in Schneider's *Drosophila* Medium (Invitrogen, San Deigo, CA) supplemented with 10% fetal bovine serum (FBS) and 1% penicillin/streptomycin. Transfection of the S2 cells was performed using Fugene6 (Roche, Indianapolis, IN) according to the manufacturer's protocol.

### Immunoprecipitations and Western analysis

At 36–48 h post-transfection, the cells were collected, washed with ice-cold PBS, and harvested in lysis buffer (20 mM Tris-HCl, pH 8.0, 150 mM NaCl, 1% Igepal, and protease inhibitors). Clarified lysates were incubated with 15–20 µl of a 50% slurry of anti-FLAG-agarose (Sigma, St. Louis, MO) or anti-HA agarose (Roche, Indianapolis, IN) overnight at 4°C. The immune complexes were washed three times with lysis buffer and eluted with SDS sample buffer. Protein samples were separated by SDS-PAGE and transferred to 0.45 µm nylon-supported membrane nitrocellulose membranes (Whatman, Dassel, Germany). Membranes were blocked in Odyssey blocking buffer (Li-COR; Lincoln, NE) and then incubated overnight at 4°C with the indicated primary antibody. After washing with Tris-buffered saline containing 0.5% BSA and 0.1% Tween-20 (TTBS/BSA), the membranes were incubated with the appropriate flouraphore-conjugated secondary antibodies. All antibodies were diluted in TTBS/BSA. Bound antibodies were visualized and analyzed using the Odyssey Infrared Imaging system and Odyssey software (LI-COR, Lincoln, NE).

### Immunostaining of imaginal discs

Third instar larva were examined and isolated under a fluorescent microscope, according to the presence of the chromosome balancer with actin-GFP. Flies carrying either the *UAS* element (*UAS-Tap42^RNAi^*) or *Gal4* driver alone were used as controls throughout this study, unless otherwise noted. Immunofluorescent staining of wing discs was performed using a previously described protocol [44]. Briefly, wing discs were dissected from wandering 3^rd^ instar larva and fixed with 4% paraformaldehyde. After washing two times with PBS, the discs was permeabilized in PBT (PBS containing 0.3% Triton X-100) and then incubated with blocking buffer containing 10% horse serum. The permeabilized wing discs were incubated with the indicated primary antibodies followed by incubation with the appropriate fluorophore-conjugate secondary antibodies. Cell nuclei were contrast stained using either DAPI or TO-PRO3 (1∶1000; Invitrogen, Carlsbad, CA) before mounting to a glass plate. The samples were then subjected to fluorescent (confocal) microscopy. All pictures were analyzed using the Zeiss LSM Image Browser software.

### TUNEL staining of wing discs

Wing discs were washed with PBS, fixed with 4% paraformaldehyde, and permeabilized in PBT. Cell apoptosis in the wing discs was visualized using the *In Situ* Cell Death Detection Kit, TMR Red (Roche, Indianapolis, IN) and confocal microscopy following the manufacturer's protocol.

## Supporting Information

Figure S1 **Fate map of wing imaginal disc from 3^rd^ instar larvae.** Schematic of 3^rd^ instar larva *Drosophila* wing imaginal disc. Regions of the wing disc that develop into the future adult notum, wing hinge, and wing are indicated. Demarcated on the DP layer (left) are blue dashed lines representing the anterior/posterior (A/P) and dorsal/ventral (D/V) boundaries that run from top to bottom and left to right, respectively. A lateral view (middle) highlights the closely associated DP and PE layers that make up the wing disc. Within the PE layer (right) is a subpopulation of PE cells located near the PE/DP boundary that have been defined as medial edge cells (red).
(TIF)

Figure S2 ***Tap42^RNAi^*** **induces pleiotrophic defects that include eclosion failure and necrosis of leg joints.** Flies expressing *Tap42^RNAi^* in the *ap* domain failed to escape from the shell after eclosion, leading to their eventual death (A). Necrosis in the joints of the 1^st^ leg was observed in some flies (red arrows, B). Genotypes: (A & B) *ap-Gal4/UAS-Tap42^RNAi^; +/+*.
(TIF)

Figure S3 **Expression of dominant-negative BSK in the** ***ap*** **gene domain fails to rescue** ***Tap42^RNAi^*** **-induced thorax cleft.** Expression of dominant-negative BSK (BSK.DN) by *ap-Gal4* induced a cleft phenotype in the notum without affecting the scutum (A). The thorax cleft phenotype induced by *Tap42^RNAi^* was not rescued by expression of *BSK.DN* (compare B with Fig. 6-B1). Instead, the cleft phenotype worsened as noted by the failure of the scutum to develop correctly. Genotypes: (A) *+/ap-Gal4; +/UAS-Bsk.DN.* (B) *ap-Gal4/UAS-Tap42^RNAi^*; *+/UAS-Bsk.DN*.
(TIF)

Figure S4 **Thorax phenotype is rescued by** ***Tap42^WT^*** **but not** ***Tap42^ED^*** **expression in the** ***pnr*** **gene domain.** Introduction of *Tap42^WT^* (B) but not *Tap42^ED^* (C) in the *pnr* domain rescued the defects associated with silencing of *Tap42* in the same domain (A). Expression of *Tap42^WT^* (D) or *Tap42^ED^* (E) with *pnr-Gal4* driver yielded no obvious thorax phenotype. Genotypes: (A) *UAS-Tap42^RNAi^/+; pnr-Gal4/+*. (B) *UAS-Tap42^RNAi^/+; pnr-Gal4/ UAS-Tap42^WT^*. (C) *UAS-Tap42^RNAi^/+; pnr-Gal4/UAS-Tap42^ED^*. (D) *+/+; pnr-Gal4/UAS-Tap42^WT^*. (E) *+/+; pnr-Gal4/UAS-Tap42^ED^*.
(TIF)

Table S1 Protein phosphatase subunit orthologues of PP2A family members in human, *Drosophila*, and yeast.
(DOCX)
