# Supplementary figures and images for: Essential Roles of the Tap42-Regulated Protein Phosphatase 2A (PP2A) Family in Wing Imaginal Disc Development of Drosophila melanogaster
Source: PLoS One. 2012 Jun 6;7(6):e38569. doi: 10.1371/journal.pone.0038569 (PMC3368869; doi:10.1371/journal.pone.0038569)

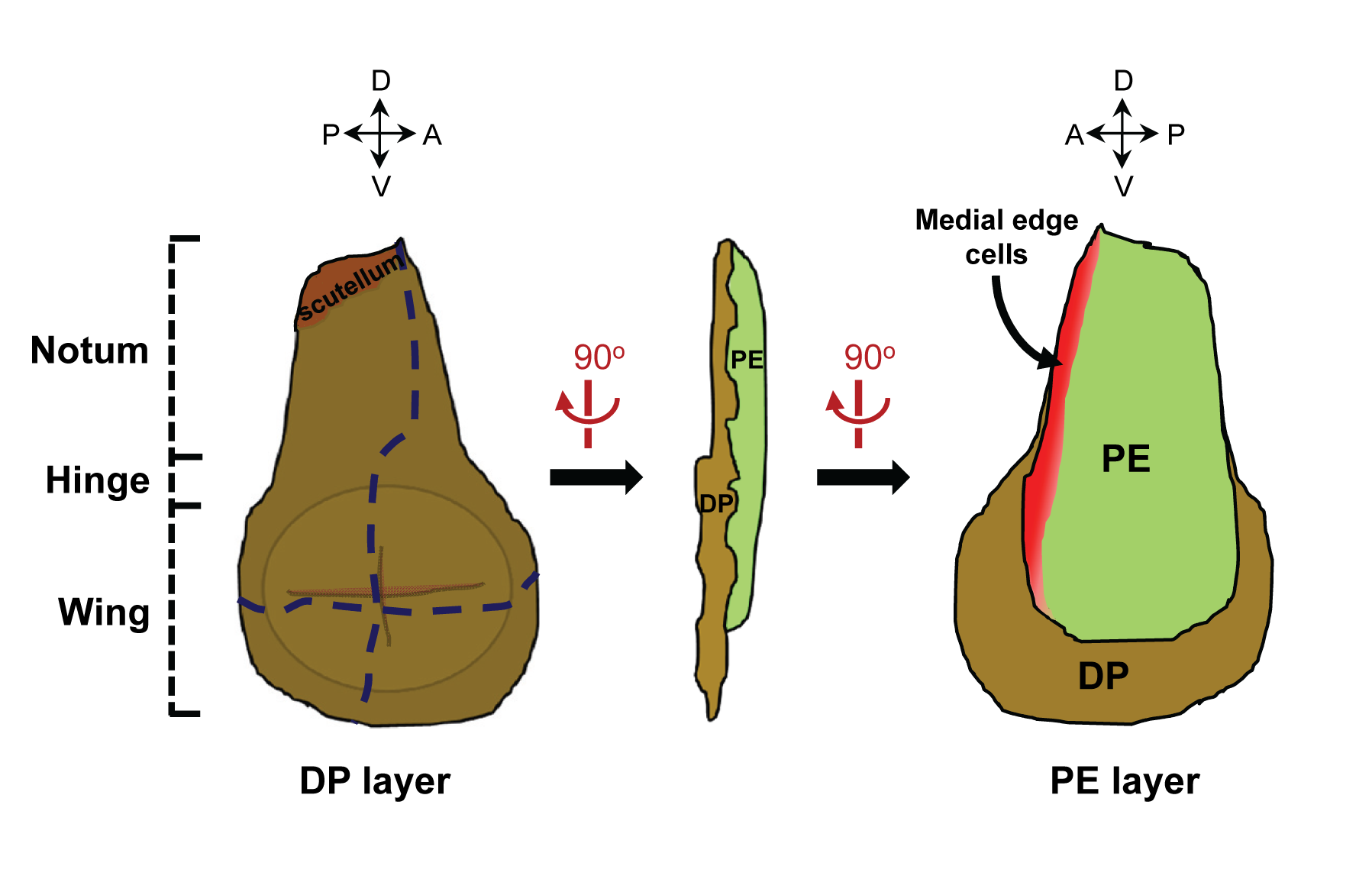

Supplement: Figure S1 — Fate map of wing imaginal disc from 3rd instar larvae. Schematic of 3rd instar larva Drosophila wing imaginal disc. Regions of the wing disc that develop into the future adult notum, wing hinge, and wing are indicated. Demarcated on the DP layer (left) are blue dashed lines representing the anterior/posterior (A/P) and dorsal/ventral (D/V) boundaries that run from top to bottom and left to right, respectively. A lateral view (middle) highlights the closely associated DP and PE layers that make up the wing disc. Within the PE layer (right) is a subpopulation of PE cells located near the PE/DP boundary that have been defined as medial edge cells (red). (TIF) [file pone.0038569.s001.tif]

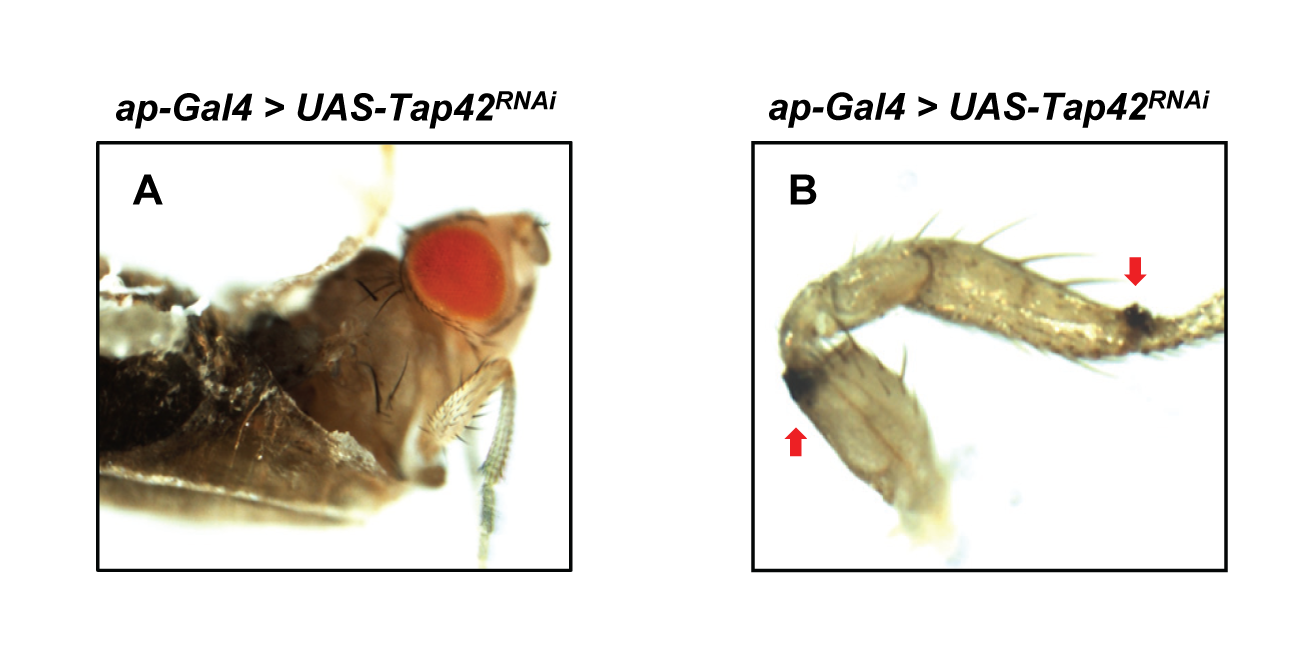

Supplement: Figure S2 — Tap42RNAi induces pleiotrophic defects that include eclosion failure and necrosis of leg joints. Flies expressing Tap42RNAi in the ap domain failed to escape from the shell after eclosion, leading to their eventual death (A). Necrosis in the joints of the 1st leg was observed in some flies (red arrows, B). Genotypes: (A & B) ap-Gal4/UAS-Tap42RNAi; +/+. (TIF) [file pone.0038569.s002.tif]

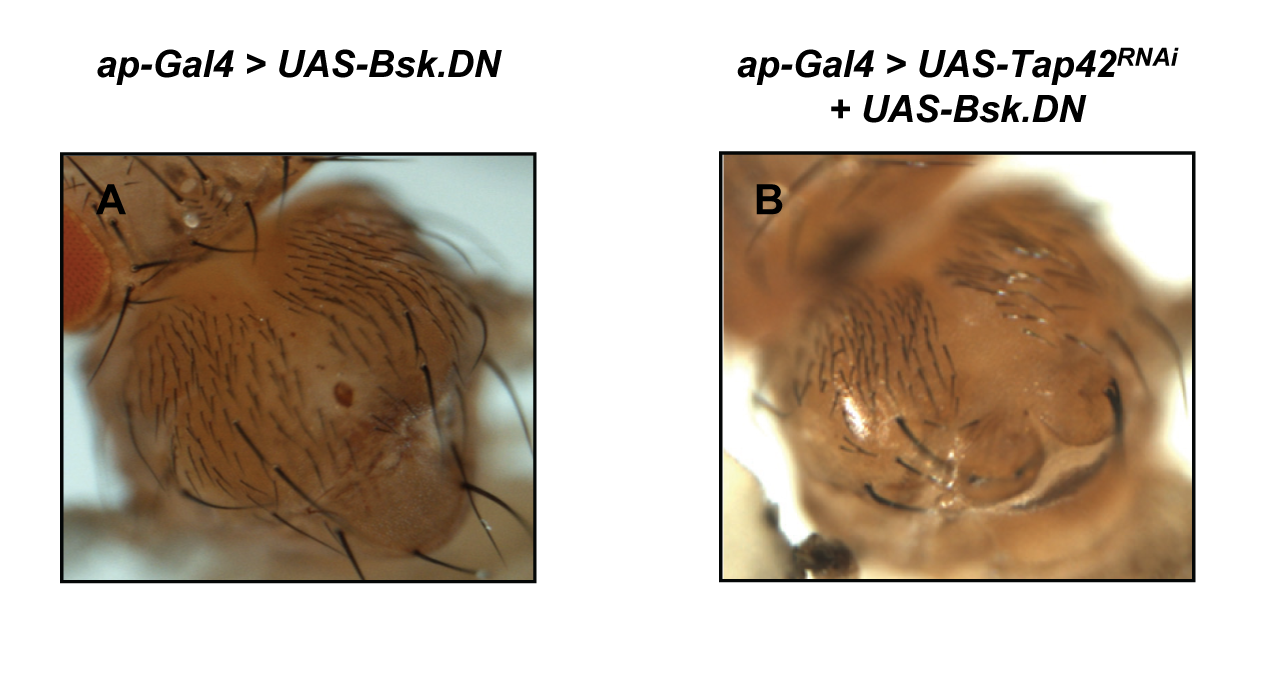

Supplement: Figure S3 — Expression of dominant-negative BSK in the ap gene domain fails to rescue Tap42RNAi -induced thorax cleft. Expression of dominant-negative BSK (BSK.DN) by ap-Gal4 induced a cleft phenotype in the notum without affecting the scutum (A). The thorax cleft phenotype induced by Tap42RNAi was not rescued by expression of BSK.DN (compare B with Fig. 6-B1). Instead, the cleft phenotype worsened as noted by the failure of the scutum to develop correctly. Genotypes: (A) +/ap-Gal4; +/UAS-Bsk.DN. (B) ap-Gal4/UAS-Tap42RNAi; +/UAS-Bsk.DN. (TIF) [file pone.0038569.s003.tif]

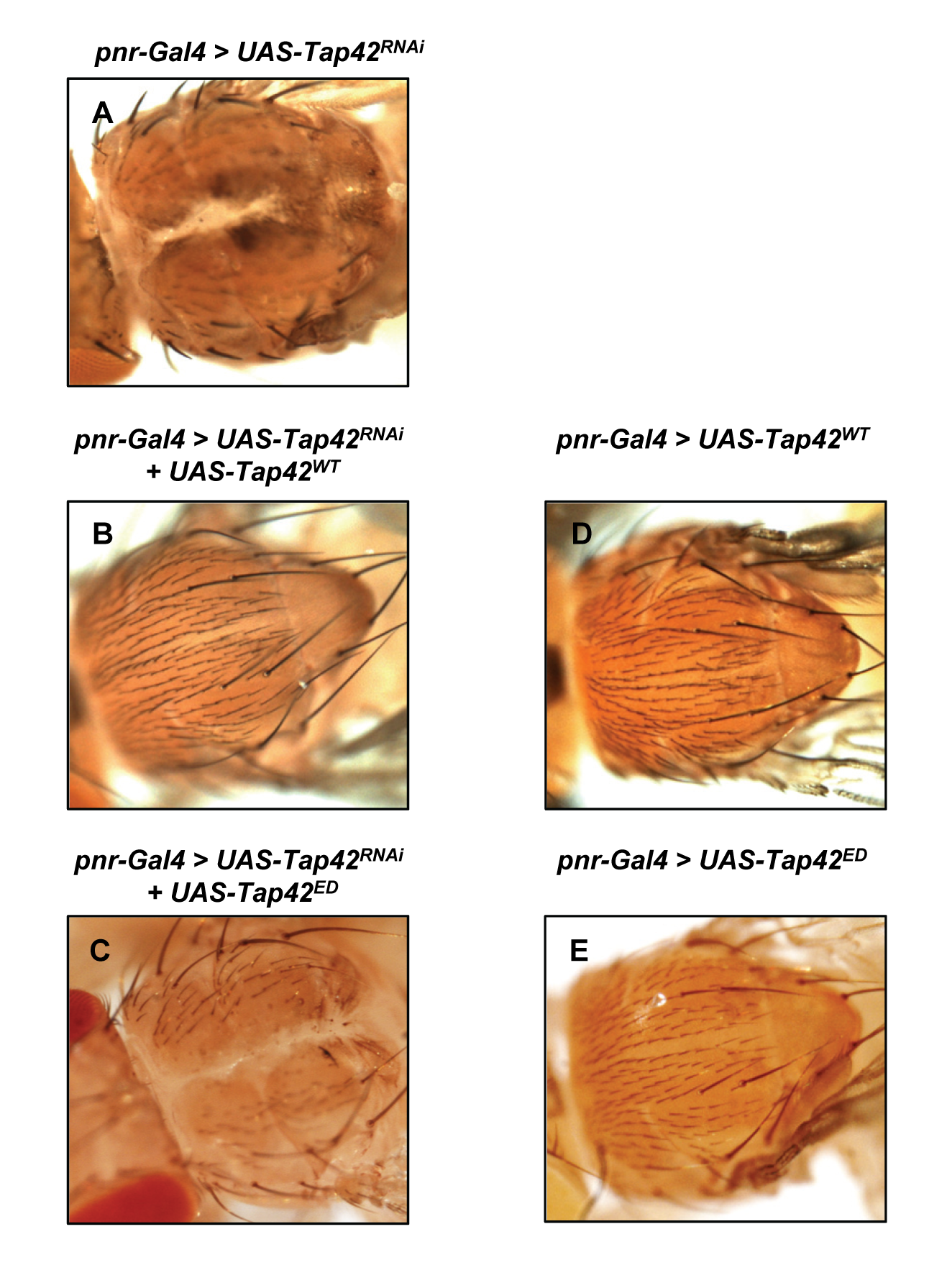

Supplement: Figure S4 — Thorax phenotype is rescued by Tap42WT but not Tap42ED expression in the pnr gene domain. Introduction of Tap42WT (B) but not Tap42ED (C) in the pnr domain rescued the defects associated with silencing of Tap42 in the same domain (A). Expression of Tap42WT (D) or Tap42ED (E) with pnr-Gal4 driver yielded no obvious thorax phenotype. Genotypes: (A) UAS-Tap42RNAi/+; pnr-Gal4/+. (B) UAS-Tap42RNAi/+; pnr-Gal4/ UAS-Tap42WT. (C) UAS-Tap42RNAi/+; pnr-Gal4/UAS-Tap42ED. (D) +/+; pnr-Gal4/UAS-Tap42WT. (E) +/+; pnr-Gal4/UAS-Tap42ED. (TIF) [file pone.0038569.s004.tif]
