# Supplementary material for: Essential Roles of the Tap42-Regulated Protein Phosphatase 2A (PP2A) Family in Wing Imaginal Disc Development of Drosophila melanogaster
Source: PLoS One. 2012 Jun 6;7(6):e38569. doi: 10.1371/journal.pone.0038569 (PMC3368869; doi:10.1371/journal.pone.0038569)
Supplement: Table S1 — Protein phosphatase subunit orthologues of PP2A family members in human, Drosophila, and yeast. (DOCX) [file pone.0038569.s005.docx]

| **Human** | ***Drosophila*** | **Yeast ^a^** |
| --- | --- | --- |
| **Catalytic subunits of PP2A family member** |  |  |
| PP2Ac(α or β) | Mts; CG7109 | PPH21 or 22 |
| PP4c | PP4-19C; CG32505 | PPH3 |
| PP6c | PPV; CG12217 | SIT4 |
| **PP2A canonical regulatory subunits** |  |  |
| PP2A-A; PR65(α or β) | PP2A-29B; CG17291 | TPD3 |
| PP2A-B; B55/PR55(α,β,γ or δ) | Twins(Tws); CG6235 | CDC55 |
| PP2A-B'; B56/PR61(α,β,γ ,δ or ε) | PP2A-B'; Widerborst(Wdb); CG5643 | RTS1 |
| PP2A-B''; PR72, PR130, PR59 or PR48 | PP2A-B''; PP2-PR72; CG4733 |  |
| PP2A-B'''; PR93/PR110 |  |  |
| **PP4 canonical regulatory subunits** |  |  |
| PP4R1 |  |  |
| PP4R2 | PPP4R2r; CG2890 | YBL046w/PSY4P |
| PP4R3(A,B); SMEK(1, 2) | R3; Flfl(Falafel); CG9351 | PSY2P |
| PP4R4; KIAA1622; PP4-A |  |  |
| **PP6 canonical regulatory subunits** |  |  |
| PP6R1 | CG10289 | SAP155 |
| PP6R2 |  | SAP185 |
| PP6R3 |  | SAP190 |
| **Atypical regulatory subunits for PP2A family members** |  |  |
| α4/alpha4/IGBP1 | Tap42; CG31852 | TAP42 |
| Tip/TIPRL1 | CG9578 | TIP41 |

1. *S.cerevisiae*
